# Reliability and validity of a nutrition and physical activity environmental self-assessment for child care

**DOI:** 10.1186/1479-5868-4-29

**Published:** 2007-07-05

**Authors:** Sara E Benjamin, Brian Neelon, Sarah C Ball, Shrikant I Bangdiwala, Alice S Ammerman, Dianne S Ward

**Affiliations:** 1 Department of Ambulatory Care and Prevention, Harvard Medical School and Harvard Pilgrim Health Care, Boston, MA USA; 2 Department of Biostatistics, School of Public Health, University of North Carolina at Chapel Hill, Chapel Hill, NC USA; 3 Center for Health Promotion and Disease Prevention, University of North Carolina at Chapel Hill, Chapel Hill, NC USA; 4 Department of Nutrition, Schools of Public Health and Medicine, University of North Carolina at Chapel Hill, Chapel Hill, NC USA

## Abstract

**Background:**

Few assessment instruments have examined the nutrition and physical activity environments in child care, and none are self-administered. Given the emerging focus on child care settings as a target for intervention, a valid and reliable measure of the nutrition and physical activity environment is needed.

**Methods:**

To measure inter-rater reliability, 59 child care center directors and 109 staff completed the self-assessment concurrently, but independently. Three weeks later, a repeat self-assessment was completed by a sub-sample of 38 directors to assess test-retest reliability. To assess criterion validity, a researcher-administered environmental assessment was conducted at 69 centers and was compared to a self-assessment completed by the director. A weighted kappa test statistic and percent agreement were calculated to assess agreement for each question on the self-assessment.

**Results:**

For inter-rater reliability, kappa statistics ranged from 0.20 to 1.00 across all questions. Test-retest reliability of the self-assessment yielded kappa statistics that ranged from 0.07 to 1.00. The inter-quartile kappa statistic ranges for inter-rater and test-retest reliability were 0.45 to 0.63 and 0.27 to 0.45, respectively. When percent agreement was calculated, questions ranged from 52.6% to 100% for inter-rater reliability and 34.3% to 100% for test-retest reliability. Kappa statistics for validity ranged from -0.01 to 0.79, with an inter-quartile range of 0.08 to 0.34. Percent agreement for validity ranged from 12.9% to 93.7%.

**Conclusion:**

This study provides estimates of criterion validity, inter-rater reliability and test-retest reliability for an environmental nutrition and physical activity self-assessment instrument for child care. Results indicate that the self-assessment is a stable and reasonably accurate instrument for use with child care interventions. We therefore recommend the Nutrition and Physical Activity Self-Assessment for Child Care (NAP SACC) instrument to researchers and practitioners interested in conducting healthy weight intervention in child care. However, a more robust, less subjective measure would be more appropriate for researchers seeking an outcome measure to assess intervention impact.

## Background

Despite concerted efforts, rates of overweight among children continue to rise [1-5]. In the United States, 26.2% of 2- to 5-year old children were classified as either overweight or at risk for overweight [1]. Even in childhood, overweight is associated with a variety of deleterious health outcomes that can include Type II diabetes mellitus [6,7], hypertension and hyperlipidemia [7,8], asthma and sleep apnea [9], early maturation, and psychosocial stress [10].

Exact causes of childhood overweight are still unknown, although behavioral and environmental influences are thought to play a significant role [11]. Child care settings have recently become a focus for environmental intervention efforts. A large percentage of children in the United States and abroad are in some form of child care, and duration of time in care has increased in recent years [12-16]. The 2001 National Household Education Survey found that 74% of all 3 to 6 year old children in the United States are in some form of non-parental care and 56% are in center-based child care [17], while just over half of all Canadian preschoolers attend child care [16].

A small number of studies have targeted nutrition, physical activity, and healthy weight in child care facilities [18-26]. While these studies provide some guidance for intervention, they also highlight the need to examine environmental influences on child weight. Though there are instruments to assess the home [27,28], school [29,30], and built environment [31], few measures of child care environments exist. The Early Childhood Environment Rating Scale (ECERS) [32] and the Infant and Toddler Environment Rating Scale (ITERS) [33], developed by the University of North Carolina at Chapel Hill Frank Porter Graham Child Development Center, include a small number of nutrition and physical activity assessment questions, but were not developed to promote healthy weight in children. Moreover, the instruments were designed to be administered by an outside rater, and are often tied to a regulatory or licensing assessment. Thus, we developed a child care-directed assessment that allows child care providers to evaluate their facility's nutrition and physical activity environments. The purpose of this paper is to report results from reliability and validity testing of a nutrition and physical activity self-assessment instrument for use in child care environments.

## Methods

### Development of the Self-Assessment Instrument

The self-assessment instrument [see Additional file 1] was developed for the Nutrition and Physical Activity Self-Assessment for Child Care (NAP SACC) intervention. The NAP SACC intervention was designed to allow child care facilities to self-assess their nutrition and physical activity environments, select areas for improvement, and make environmental changes with the help of a local health consultant (NAP SACC Consultant). Trained NAP SACC Consultants provided technical assistance and support for environmental improvements at child care facilities.

To develop the NAP SACC self-assessment instrument, we conducted a thorough review of nutrition and physical activity standards and recommendations for children ages 2 to 5 years and child care. In addition, we searched the scientific literature for nutrition and physical activity recommendations for young children. In-depth results of this review can be found elsewhere [34]. Based on these reviews, we developed key nutrition and physical activity areas of focus. Key NAP SACC nutrition areas of focus included: Fruits and Vegetables; Fried Foods and High Fat Meats; Beverages; Menus and Variety; Meals and Snacks; Foods Outside of Regular Meals and Snacks; Supporting Healthy Eating; Nutrition Education for Children, Parents and Staff; and Nutrition Policy. Key NAP SACC physical activity areas of focus included: Active Play and Inactive Time; TV Use and TV Viewing; Play Environment; Supporting Physical Activity; Physical Activity Education for Children, Parents, and Staff; and Physical Activity Policy. The self-assessment instrument included 38 nutrition and 18 physical activity questions that had a demonstrated relationship to childhood overweight, or were likely contributors to an unhealthy environment. Each question had four possible response options ranging from minimum standard to best practice. The NAP SACC self-assessment instrument and accompanying intervention were developed based on aspects of Social Cognitive Theory (SCT), which describes individual behaviors as stemming from environmental influences, and identifies several crucial factors that influence behavior change including observational learning, self-efficacy, environment, reinforcement, and reciprocal determinism [35]. In addition to SCT, the socio-ecological framework helps to describe the relationship between an individual and the environment [36]. Additional information on the NAP SACC intervention and further description of the nutrition and physical activity areas of focus for the self-assessment instrument are described elsewhere [34,37].

### Sample

Ninety-six child care centers from across North Carolina were recruited to participate in the NAP SACC intervention. Thirty-two Child Care Health Consultants (CCHC) were recruited to serve as NAP SACC Consultants for the project, and were then asked to provide a list of child care centers from their local area. Employed in a number of states, CCHC are typically Registered Nurses who provide health consultation to child care facilities [38]. Child care facilities were eligible to participate if they had at least 15 children enrolled and were classified as a child care center and not a family child care home (served more than 5 preschool-aged children). Child care centers that met eligibility requirements received a telephone call from the study coordinator inviting them to participate in the research study. Of the 96 centers that enrolled in the study, 70 were randomly assigned to a treatment arm that included completion of the self-assessment instrument, while the remaining 26 served as control centers and did not complete the self-assessment instrument.

Characteristics of the child care centers used for each analysis are described in Table 1. Descriptive personal information was not collected for child care center directors or staff members. All procedures were approved by the University of North Carolina – Chapel Hill Biomedical Institutional Review Board, and all participants gave written informed consent to participate in the study.

**Table 1:** Characteristics of the Child Care Centers

**Child Care Center Characteristic**	**Sample Mean (SD, Range) **N = 69
Years in operation	17.0 (11.53, 1–45)
Number of children enrolled	79.4 (53.6, 12–230)
Number of classrooms	6.0 (3.3, 1–17)
Number of staff members	16.1 (13.3, 2–85)
CACFP participant (%)	81.2 (39.4)
NAEYC accredited (%)	6 (2.8)
African American or Black children (%)	20.5 (26.8, 0–98)
Asian or Pacific Islander children (%)	3.6 (1.7, 0–10)
Native American children (%)	5.8 (21.2, 0–100)
White children (%)	61.7 (33.7, 0–100)
More than one race children (%)	2.8 (6.1, 0–25)
Ethnicity Hispanic or Latino/a children (%)	3.5 (7.5, 0–48)

CACFP = Child and Adult Care Adult Food Program

NAEYC = National Association for the Education of Young Children

### Reliability Testing

Test-retest and inter-rater reliability testing was conducted on the NAP SACC self-assessment instrument to assess the ability of the instrument to yield consistent results with repeat administration and with multiple raters. Two self-assessment instruments were completed by child care center directors over a three week period of time, which is a method consistent with other studies that measured test-retest reliability [39,40]. To assess inter-rater reliability, the child care center director and two additional staff members were asked to completed the initial self-assessment instrument concurrently, but independently. In 50 centers, two additional staff members completed the self-assessment, while in 9 child care centers only one additional staff member completed the self-assessment instrument. Thus, 50 triad and 9 dyads were created to assess inter-rater reliability.

Self-assessment instruments were mailed to all 70 child care center directors, and 69 (99%) returned the instrument. Three weeks after the initial self-assessment instruments were received, center directors were asked to complete a second self-assessment instrument to assess test-retest reliability. Of the 69 center directors that completed the initial instrument, 38 (55%) returned the second self-assessment instrument.

### Validity Testing

#### NAP SACC Self-Assessment Instrument

Criterion validity of the NAP SACC self-assessment instrument was evaluated for this project. Face, although its worth has been contested [41], and content validity were reasonably established in a comprehensive literature and resource review that was conducted prior to the development of the self-assessment instrument [34]. In addition, construct validity was assessed in a national expert review that took place in January through April of 2004. Overall, the reviewers found the instrument to be an accurate and comprehensive measure of the nutrition and physical activity child care center environment; however, over the course of three months, a number of revisions were made to the instrument based on reviewer recommendations.

#### The Environment and Policy Assessment and Observation (EPAO) System

To assess criterion validity, the NAP SACC self-assessment instrument was compared to observation and document reviews at the child care center. The Environment and Policy Assessment and Observation (EPAO) system was developed to objectively assess the diet and physical activity environment of child care centers (Ward, 2006, unpublished data). A main component of the EPAO is the one-day observation conducted at the child care center. The observation sections of the EPAO were divided into 7 sections: 1. Eating occasions-Foods; 2. Eating Occasions-Beverages; 3. Eating Occasions-Staff Behaviors; 4. Physical Activity-Child Behaviors; 5. Sedentary Activities-Child; 6. Physical Activity-Staff Behaviors; and 7. Center Environment. Additionally, completion of the EPAO included a review of lesson plans, fundraising documents, menus, parent handbooks, staff training documents, playground safety check policies, physical activity and nutrition education training documents, and overall nutrition and physical activity policies.

A group of five field observers were trained during a one-day intensive workshop by the developers of the EPAO system. One observer held a bachelor's degree in nutrition and four had completed or were in the process of completing a master's or doctorate degree in a health-related field. Training included a review of the EPAO system components as well as lessons on general observation techniques, types of play equipment and space, instruction and demonstration of record keeping, and an overview of general child care center rules, regulations, and state mandates. Additionally, each field observer completed a practice observation in a child care center. Prior to beginning data collection, each field observer was required to attain 85% agreement with the gold standard observer who assisted in the development the EPAO. Inter-rater reliability testing was also conducted throughout the data collection period and all field observers periodically underwent retraining to prevent observer drift.

The EPAO was used as the gold standard comparison for the NAP SACC self-assessment instrument. The EPAO, however, could not be used to assess validity for 8 of the 38 (21%) nutrition and 4 of the 18 (22%) physical activity questions (Table 2). These questions required more than a one-day observation in the child care facility and typically assessed practices that may occur 1 or 2 times per year. Moreover, documentation was not available for these practices and therefore the information could not be ascertained through the document review (e.g., PA5D Physical activity education is offered to parents: rarely or never; less than 1 time per year; 1 time per year, 2 times per year).

**Table 2:** Validity Measures Using Weighted Kappa Test Statistics and Percent Agreement

**Self-Assessment Question**	**Validity**			
	**Validation Method**	**Kappa**	**95% CI**	**Percent Agreement**

**Nutrition**				

N1A. Fruit (not juice)	Document Review	0.31	0.15–0.47	43.8
N1B. Fresh, frozen, or canned in juice fruit	**--**	---	---	---
N1C. 100% fruit juice	Document Review	0.23	0.06–0.41	42.2
N1D. Vegetables (not including fried potatoes)	Document Review	0.06	-0.10–0.02	47.6
N1E. Dark green, red, orange, or yellow vegetables	Document Review	0.08	-0.08–0.24	12.9
N1F. Vegetables and added fat	---	---	---	---
N2A. Fried or pre-fried meats	Document Review	0.19	-0.03–0.40	59.4
N2B. Fried or pre-fried potatoes	Document Review	0.22	0.04–0.40	53.8
N2C. High fat meats	Document Review	0.06	-0.03–0.15	26.2
N2D. Lean meats	Document Review	0.13	-0.04–0.30	41.5
N3A. Outdoor drinking water	Observation	0.17	-0.01–0.35	33.3
N3B. Indoor drinking water	Observation	0.40	0.23–0.58	60.0
N3C. Sugar-sweetened beverages	Document Review	0.26	-0.12–0.64	93.7
N3D. Type of milk for children ages 2 and older	Observation	0.73	0.59–0.88	82.1
N3E. Soft-drink vending machines	Observation	0.78	0.67–0.90	83.1
N4A. Cycle menu length	Document Review	0.06	-0.17–0.29	41.8
N4B. Whole grain, high fiber	Document Review	0.03	0.00–0.05	26.6
N4C. Introduction of new foods	Observation	---	---	---
N4D. Foods from other cultures	Document Review	0.25	0.10–0.41	56.1
N5A. Satiety	Observation	0.18	0.02–0.34	36.1
N5B. Hunger	Observation	-0.01	-0.12–0.10	27.5
N5C. Encouraging children to eat	Observation	0.08	0.02–0.14	30.2
N5D. Sweets, high fat, high salt	Document Review	0.03	0.00–0.06	17.2
N5E. Food as reward	Observation	0.33	-0.08–0.73	92.5
N5F. Food used to control behavior	Observation	0.00	0.00–0.00	87.9
N6A. Parent guidelines for holidays or celebrations	Document Review	0.35	0.19–0.50	47.5
N6B. Holidays and celebrations	Document Review	---	---	---
N6C. Fundraising	Document Review	0.23	-0.09–0.55	33.3
N7A. Children and staff sit together for meals	Observation	0.22	0.10–0.35	32.8
N7B. Meals served family style	Observation	0.55	0.30–0.80	82.1
N7C. Staff consume the same foods and drinks as children	Observation	0.32	0.17–0.47	47.5
N7D. Staff consume less healthy foods in front of children	Observation	0.11	-0.11–0.34	55.9
N7E. Staff talk with children about healthy foods	Observation	0.04	-0.07–0.14	22.5
N8A. Training opportunities on nutrition for staff	---	---	---	---
N8B. Nutrition training provided by qualified professional	---	---	---	---
N8C. Staff provide nutrition education for children	---	---	---	---
N8D. Nutrition education offered to parents	---	---	---	---
N9A. Written policy on nutrition and food service	Document Review	0.76	0.60–0.92	88.1

**Physical Activity**				

PA1A. Active (free) play time	Observation	0.12	-0.05–0.30	44.6
PA1B. Structured physical activity	Observation	0.34	0.10–0.59	59.7
PA1C. Outdoor active play	Observation	0.16	0.02–0.31	52.2
PA1D. PA as punishment	Observation	0.07	-0.04–0.17	36.4
PA1E. Sedentary time	Observation	0.00	0.00–0.00	0.0
PA2A. Presence of television	Observation	0.48	0.30–0.65	67.2
PA2B. TV, videos, video games	Observation	0.60	0.42–0.77	75.4
PA3A. Fixed play equipment	Observation	0.77	0.63–0.90	83.6
PA3B. Equipment safety checks	Observation	0.14	-0.14–0.24	65.9
PA3C. Portable play equipment	Observation	0.45	0.29–0.60	59.7
PA3D. Indoor play space	Observation	0.18	0.03–0.32	40.3
PA4A. Staff join in active play	Observation	0.59	0.43–0.75	69.7
PA4B. Support for PA	Observation	0.28	0.15–0.42	35.4
PA5A. Training opportunities on PA for staff	---	---	---	---
PA5B. PA training by qualified professional	---	---	---	---
PA5C. Staff provide PA education for children	---	---	---	---
PA5D. PA education offered to parents	---	---	---	---
PA6A. Written policy on PA	Document Review	0.79	0.63–0.95	90.6

PA = Physical activity

Sixty-nine child care centers were visited by field observers to assess the nutrition and physical activity environments using the EPAO. Immediately following this visit, child care center directors and staff were asked to complete the NAP SACC self-assessment instrument. Results from the EPAO were compared to the self-assessment instrument completed by the center directors to assess criterion validity.

### Statistical Analyses

The test-retest reliability comparison between time 1 and time 2 was conducted on self-assessment instruments from 38 child care center directors. Inter-rater reliability was calculated using time 1 data from 59 child care centers (9 child care center director/teacher dyads and 50 child care center director/teacher triads). The proportion in exact agreement (percent agreement) and a weighted kappa statistic were calculated to assess overall agreement for each question on the self-assessment instrument. A weighted kappa statistic [42] was calculated to assess agreement for each question on the self-assessment instrument compared to the EPAO using data from the 69 child care centers. Percent agreement was also calculated for each question.

## Results

### Reliability

Results for all reliability measures are reported in Table 3. Test-retest reliability of the self-assessment instrument yielded kappa statistics that ranged from 0.07 to 1.00 across all questions. The least reliable question asked how often nutrition education was provided to parents of the children in care (N8D). For inter-rater reliability, kappa statistics ranged from 0.20 to 1.00 across all questions. The question that yielded the lowest kappa statistic asked how often fat was added to cooked vegetables (N1F). The most reliable question for both test-retest and inter-rater reliability yielded a kappa of 1.00 for the question that assessed how often food was used to control behavior (N5F). The inter-quartile ranges for test-retest and inter-rater reliability were 0.27 to 0.45 and 0.45 to 0.63, respectively. When percent agreement was calculated, questions ranged from 34.29 to 100.00 for test-retest reliability and 52.62 to 100.00 for inter-rater reliability.

**Table 3:** Reliability Measures Using Weighted Kappa Test Statistics and Percent Agreement

**Self-Assessment Question**	**Test-Retest Reliability**			**Inter-Rater Reliability**		
	**Kappa**	**95% CI**	**Percent Agreement**	**Kappa**	**95% CI**	**Percent Agreement**

**Nutrition**						

N1A. Fruit (not juice)	0.35	0.20–0.51	57.0	0.54	0.30–0.79	68.4
N1B. Fresh, frozen, or canned in juice fruit	0.30	0.08–0.51	73.4	0.40	0.06–0.73	76.3
N1C. 100% fruit juice	0.44	0.30–0.58	60.2	0.65	0.44–0.86	75.7
N1D. Vegetables (not including fried potatoes)	0.39	0.23–0.55	65.1	0.30	0.02–0.58	61.1
N1E. Dark green, red, orange, or yellow vegetables	0.09	-0.05–0.24	50.0	0.35	0.11–0.59	58.3
N1F. Vegetables and added fat	0.38	0.23–0.52	55.3	0.20	-0.06–0.47	54.3
N2A. Fried or pre-fried meats	0.27	0.11–0.42	62.1	0.28	0.05–0.51	62.2
N2B. Fried or pre-fried potatoes	0.42	0.27–0.58	69.8	0.59	0.34–0.83	78.4
N2C. High fat meats	0.31	0.16–0.46	62.6	0.37	0.09–0.62	67.5
N2D. Lean meats	0.28	0.14–0.43	53.4	0.39	0.15–0.63	55.6
N3A. Outdoor drinking water	0.57	0.45–0.68	60.8	0.63	0.42–0.83	69.4
N3B. Indoor drinking water	0.41	0.26–0.57	66.4	0.67	0.47–0.87	73.7
N3C. Sugar-sweetened beverages	0.48	0.10–0.87	96.2	0.85	0.54–1.00	97.3
N3D. Type of milk for children ages 2 and older	0.75	0.64–0.87	83.3	0.86	0.74–0.98	86.9
N3E. Soft-drink vending machines	0.86	0.79–0.94	89.8	0.90	0.79–1.00	92.1
N4A. Cycle menu length	0.59	0.44–0.73	71.8	0.60	0.36–0.84	78.9
N4B. Whole grain, high fiber	0.39	0.25–0.53	53.3	0.39	0.16–0.62	52.6
N4C. Introduction of new foods	0.22	0.08–0.37	50.5	0.48	0.26–0.70	60.5
N4D. Foods from other cultures	0.24	0.08–0.40	53.3	0.49	0.29–0.70	60.5
N5A. Satiety	0.33	0.17–0.49	54.6	0.56	0.36–0.75	60.5
N5B. Hunger	0.14	0.00–0.28	34.3	0.61	0.42–0.80	63.2
N5C. Encouraging children to eat	0.26	0.10–0.41	59.6	0.45	0.18–0.72	68.4
N5D. Sweets, high fat, high salt	0.29	0.13–0.44	65.1	0.59	0.35–0.83	79.0
N5E. Food as reward	0.19	-0.09–0.46	89.0	0.32	0.06–0.58	94.7
N5F. Food used to control behavior	1.00	1.00–1.00	100.0	1.00	1.00–1.00	100.0
N6A. Parent guidelines for holidays or celebrations	0.41	0.27–0.55	54.5	0.48	0.23–0.72	61.1
N6B. Holidays and celebrations	0.31	0.16–0.46	48.6	0.54	0.34–0.74	60.5
N6C. Fundraising	0.23	0.09–0.36	42.0	0.42	0.16–0.68	61.8
N7A. Children and staff sit together for meals	0.60	0.49–0.71	62.4	0.68	0.51–0.85	68.4
N7B. Meals served family style	0.77	0.67–0.88	81.5	0.85	0.73–0.97	86.8
N7C. Staff consume the same foods and drinks as children	0.51	0.40–0.62	54.1	0.40	0.18–0.62	60.5
N7D. Staff consume less healthy foods in front of children	0.36	0.18–0.53	73.8	0.45	0.17–0.73	757
N7E. Staff talk with children about healthy foods	0.23	0.08–0.37	46.3	0.58	0.39–0.77	68.4
N8A. Training opportunities on nutrition for staff	0.30	0.14–0.45	51.4	0.50	0.28–0.71	56.8
N8B. Nutrition training provided by qualified professional	0.33	0.19–0.47	44.0	0.50	0.29–0.72	60.5
N8C. Staff provide nutrition education for children	0.22	0.08–0.36	41.5	0.56	0.35–0.77	60.5
N8D. Nutrition education offered to parents	0.07	-0.11–0.24	54.9	0.29	0.01–0.57	67.6
N9A. Written policy on nutrition and food service	0.44	0.28–0.61	65.6	0.53	0.29–0.78	67.7

**Physical Activity**						

PA1A. Active (free) play time	0.41	0.27–0.56	66.1	0.55	0.32–0.78	71.1
PA1B. Structured physical activity	0.24	0.09–0.39	57.8	0.64	0.48–0.80	76.3
PA1C. Outdoor active play	0.39	0.22–0.56	75.2	0.67	0.40–0.94	89.5
PA1D. PA as punishment	0.19	0.04–0.34	48.2	0.47	0.21–0.74	72.2
PA1E. Sedentary time	0.38	0.18–0.57	77.1	0.44	0.09–0.79	78.9
PA2A. Presence of television	0.70	0.54–0.86	87.4	0.50	0.29–0.72	73.0
PA2B. TV, videos, video games	0.63	0.50–0.76	77.0	0.72	0.49–0.94	83.8
PA3A. Fixed play equipment	0.46	0.30–0.63	63.3	0.56	0.30–0.81	65.8
PA3B. Equipment safety checks	0.37	0.20–0.54	69.8	0.56	0.29–0.83	79.0
PA3C. Portable play equipment	0.34	0.20–0.48	52.3	0.60	0.42–0.78	65.8
PA3D. Indoor play space	0.31	0.16–0.47	61.5	0.85	0.68–1.00	92.1
PA4A. Staff join in active play	0.32	0.18–0.46	50.9	0.46	0.25–0.67	57.9
PA4B. Support for PA	0.17	0.04–0.31	37.1	0.62	0.43–0.81	64.9
PA5A. Training opportunities on PA for staff	0.33	0.19–0.47	44.8	0.63	0.45–0.81	60.5
PA5B. PA training by qualified professional	0.32	0.17–0.48	50.0	0.66	0.51–0.82	62.2
PA5C. Staff provide PA education for children	0.45	0.31–0.58	52.3	0.45	0.23–0.67	52.6
PA5D. PA education offered to parents	0.25	0.08–0.43	73.1	0.55	0.30–0.79	81.1
PA6A. Written policy on PA	0.37	0.19–0.54	62.4	0.71	0.50–0.93	82.4

PA = Physical activity

### Validity

Kappa statistics across all questions for validity ranged from -0.01 to 0.79, while percent agreement ranged from 0 to 93.65 (Table 2). The only question with a negative kappa, and the least valid question, asked how often child care providers assessed hunger before providing additional helpings of food to children (N5B). The most valid question with a kappa statistic of 0.79 asked about a written policy on physical activity (PA6A). Additionally, the companion nutrition policy question (N9A) yielded a kappa of 0.76. When direct observation was used to validate questions, kappa statistics ranged from -0.01 to 0.78. Questions that were validated using the document review ranged from 0.03 to 0.79. The inter-quartile range for overall validity was 0.08 to 0.34 for kappa statistics and 35.38 to 67.20 for percent agreement.

A kappa statistic, proposed by Cohen in 1960 [42], is generally a very conservative measure and takes into consideration agreement due to chance. Landis and Koch suggest the following guidelines for interpreting kappa statistics, but state clearly in their article that the guidelines are completely arbitrary: < 0 = poor agreement, 0 to 0.2 = slight agreement, 0.2 to 0.4 = fair agreement, 0.4 to 0.6 = moderate agreement, 0.6 to 0.8 = substantial agreement, and 0.8 to 1 = almost perfect agreement [43]. Applying this method for interpretation, 34% of questions for test-retest reliability, and 81% of questions for inter-rater reliability had kappa statistics greater than or equal to 0.40 (at least moderate agreement). Additionally, 25% of the questions for validity yielded kappa statistics representing at least moderate agreement.

Muñoz and Bangdiwala [44], however, conducted simulations of the behavior of kappa under different patterns of agreement, and under different proportions of agreement. The authors suggest the following alternate interpretation of the kappa statistic: < 0 = poor agreement, 0 to 0.20 = fair agreement, 0.20 to 0.45 = moderate agreement, 0.45 to 0.75 = substantial agreement, 0.75 to 1.00 = almost perfect agreement. Using this method, 89% of test-retest, 100% of inter-rater, and 52% of validity kappa statistics show at least moderate agreement (0.20 or above). We prefer to use this less arbitrary, more rigorously tested method for interpreting a kappa statistic. Table 4 presents the strength of agreement for each question for all tests.

**Table 4:** Number of Questions According to Strength of Agreement [44]

	**Test-Retest Reliability**	**Inter-Rater Reliability**	**Validity**
**Nutrition**			

N1A. Fruit (not juice)	Moderate	Substantial	Moderate
N1B. Fresh, frozen, or canned in juice fruit	Moderate	Moderate	--
N1C. 100% fruit juice	Moderate	Substantial	Moderate
N1D. Vegetables (not including fried potatoes)	Moderate	Moderate	Fair
N1E. Dark green, red, orange, or yellow vegetables	Fair	Moderate	Fair
N1F. Vegetables and added fat	Moderate	Moderate	--
N2A. Fried or pre-fried meats	Moderate	Moderate	Moderate
N2B. Fried or pre-fried potatoes	Moderate	Substantial	Fair
N2C. High fat meats	Moderate	Moderate	Fair
N2D. Lean meats	Moderate	Moderate	Fair
N3A. Outdoor drinking water	Substantial	Substantial	Moderate
N3B. Indoor drinking water	Moderate	Substantial	Moderate
N3C. Sugar-sweetened beverages	Substantial	Almost Perfect	Substantial
N3D. Type of milk for children ages 2 and older	Almost Perfect	Almost Perfect	Substantial
N3E. Soft-drink vending machines	Almost Perfect	Almost Perfect	Fair
N4A. Cycle menu length	Substantial	Substantial	Fair
N4B. Whole grain, high fiber	Moderate	Moderate	Fair
N4C. Introduction of new foods	Moderate	Substantial	--
N4D. Foods from other cultures	Moderate	Substantial	Moderate
N5A. Satiety	Moderate	Substantial	Fair
N5B. Hunger	Fair	Substantial	Poor
N5C. Encouraging children to eat	Moderate	Substantial	Fair
N5D. Sweets, high fat, high salt	Moderate	Substantial	Fair
N5E. Food as reward	Fair	Moderate	Moderate
N5F. Food used to control behavior	Almost Perfect	Almost Perfect	Poor
N6A. Parent guidelines for holidays or celebrations	Moderate	Substantial	Moderate
N6B. Holidays and celebrations	Moderate	Substantial	--
N6C. Fundraising	Moderate	Moderate	Moderate
N7A. Children and staff sit together for meals	Substantial	Substantial	Moderate
N7B. Meals served family style	Almost Perfect	Almost Perfect	Substantial
N7C. Staff consume the same foods and drinks as children	Substantial	Moderate	Moderate
N7D. Staff consume less healthy foods in front of children	Moderate	Substantial	Fair
N7E. Staff talk with children about healthy foods	Moderate	Substantial	Fair
N8A. Training opportunities on nutrition for staff	Moderate	Substantial	--
N8B. Nutrition training provided by qualified professional	Moderate	Substantial	--
N8C. Staff provide nutrition education for children	Moderate	Substantial	--
N8D. Nutrition education offered to parents	Fair	Moderate	--
N9A. Written policy on nutrition and food service	Moderate	Substantial	Almost Perfect

**Physical Activity**			

PA1A. Active (free) play time	Moderate	Substantial	Fair
PA1B. Structured physical activity	Moderate	Substantial	Moderate
PA1C. Outdoor active play	Moderate	Substantial	Fair
PA1D. PA as punishment	Fair	Substantial	Fair
PA1E. Sedentary time	Moderate	Moderate	Fair
PA2A. Presence of television	Substantial	Substantial	Substantial
PA2B. TV, videos, video games	Substantial	Substantial	Substantial
PA3A. Fixed play equipment	Substantial	Substantial	Almost Perfect
PA3B. Equipment safety checks	Moderate	Substantial	Fair
PA3C. Portable play equipment	Moderate	Substantial	Substantial
PA3D. Indoor play space	Moderate	Almost Perfect	Fair
PA4A. Staff join in active play	Moderate	Substantial	Substantial
PA4B. Support for PA	Fair	Substantial	Moderate
PA5A. Training opportunities on PA for staff	Moderate	Substantial	--
PA5B. PA training by qualified professional	Moderate	Substantial	--
PA5C. Staff provide PA education for children	Substantial	Substantial	--
PA5D. PA education offered to parents	Moderate	Substantial	--
PA6A. Written policy on PA	Moderate	Substantial	Almost Perfect

## Discussion

This paper reports on the evaluation of a self-assessment instrument designed for use with child care providers. Test-retest and inter-rater reliability, as well as criterion validity, were assessed using a weighted kappa statistic. Interpreting these data using the method proposed by Muñoz and Bangdiwala [44], overall reliability and validity of the instrument indicate it is an accurate and stable measure of the child care environment. This approach provides less arbitrary, simulation-based interpretation guidelines for the kappa test statistic, and improves upon the conventional method proposed by Landis and Koch in 1977 [43].

A limitation of the kappa statistic as a measure of concordance was demonstrated when analyzing these data. Question N5F assessed food used to control behavior, and yielded a kappa statistic of 0.00. Given that there was no variability in the scores reported on the self-assessment instrument for that question (all center directors reported a score of "4"), the weighted kappa (Cicchetti and Allison [45] weight used) was unable to yield a meaningful test statistic and therefore did not accurately represent agreement between the two measures. With the exception of this one question (N5F), responses on the NAP SACC self-assessment ranged from 1–4 for 44 of the 56 questions. For 11 of the questions, responses were limited to three of the four categories (N1B, NIE, N3C, N5C, N5D, N7D, PA1C, PA1D, PA2A, PA2B, PA3B), with variability on which response category was not selected, and in the situation described above, only one response category was selected by all respondents for one question. Percent agreement for this question (N5F) was 87.88%, which provided some indication of reasonable concordance. In this specific case, an alternate test of agreement would be more appropriate [46]. Thus, in addition to weighted kappa statistics, percent (exact) agreement is also presented for these data. Although this measure does not consider agreement due to chance, and therefore may report inflated agreement, it provided a more appropriate interpretation for question N5F and is not without overall merit.

Regardless of statistical test used, for validity testing, scores on the self-assessment instrument were higher than those on the EPAO for more than 2/3 of the questions. This was expected, given that self-report may be associated with social desirability. Child care center directors may wish to describe their center in the best possible light, which is a limitation of the self-assessment approach. The original intent of the NAP SACC self-assessment instrument, however, was to raise awareness and spark interest in the child care staff completing the instrument. Use of the instrument as a primary outcome measure for research studies is not recommended, or should be done with caution. A more objective measure, such as the EPAO may be more appropriate if researchers hope to accurately capture policies and practices at the child care facility. The EPAO, however, is not without limitations. Observation that takes place over one day will capture only those behaviors and practices that occur regularly, or happen to coincide with the day of observation. In addition, child care center staff may behave or interact differently with children in the presence of an outside observer. Repeated day observation may yield more accurate results since behaviors that happen sporadically could be observed and staff may be less likely to alter behavior after a number of observation days. In general, questions that assessed the behaviors of staff (N1D, N1E, N2C, N4A, N4B, N5B, N5C, N5D, N7E, PA1D, and PA1E) had lower kappa statistics than questions that examined more concrete outcomes. The questions that had the highest kappa statistics for both types of reliability assessed fixed, or tangible aspects of the child care center environment (N3E, N7B, N9A, PA2B, PA3A, and PA6A), although this pattern did not hold when applied to validity kappa test statistics. Review of documents (e.g., menus, lesson plans, policies) may help to supplement information gleaned from observation, but there is some evidence, however, that menus may not always accurately reflect food served at the child care center [47].

When questions on the NAP SACC instrument were broken down by category and separated by a kappa test statistic of less than .20 compared to those questions with a kappa test statistic of greater than or equal to .20, some within category patterns emerged. Questions related to staff behavior and provision of food were fairly evenly split, while questions that assessed center behavior (e.g., fundraising practices) and the overall environment tended to have more questions with a lower kappa test statistic. The category that yielded the highest percentage of kappa test statistics at or above .20 was provision of physical activity.

An additional limitation of the study is the small sample size for test-retest reliability testing, and the potential non-response bias with this sample that differs in race from the total sample. Center directors who completed a second self-assessment instrument (n = 38) were more likely to be in centers who served predominately white children, and had fewer African-American and Native American children. No differences emerged between the center staff who participated in the inter-rater reliability (n = 59) and the validity (n = 69) testing.

Despite some limitations, results for validity testing in this sample of child care centers were not without merit. Validity testing yielded kappa statistics lower than those found for reliability, but still provided evidence for reasonable agreement among the two measurement instruments. Reliability testing generally yielded higher kappa statistics, and inter-rater reliability results were slightly better than those for test-retest reliability. Raters from the same child care centers may have worked together and answered questions similarly, despite instructions to complete the self-assessment instruments independently, which is a limitation of this study. On the other hand, given that kappa statistics were excellent but not perfect, raters could be accurately reporting the same behaviors and policies seen at their child care center.

Future studies may wish to employ both an objective measure of the child care environment, as well as the self-assessment instrument pre- and post-intervention to see if the instruments perform in a similar, or parallel manner. Further assessment of the validity of the self-assessment instrument should be conducted using multiple days of observation, with less reliance on menus for documentation of actual food served. Questions with poor reliability and validity may be revised and retested, or eliminated from the final instrument.

## Conclusion

Results indicate that the self-assessment is a stable and reasonably accurate instrument for use with child care interventions. We therefore recommend the Nutrition and Physical Activity Self-Assessment for Child Care (NAP SACC) tool to researchers and practitioners interested in conducting healthy weight intervention in child care. Evaluation of its use to spark change in the child care environment is currently under study. A more robust, less subjective measure would be more appropriate for researchers seeking an outcome measure to assess intervention impact.

## Competing interests

The author(s) declare that they have no competing interests.

## Authors' contributions

SEB conceived of the study, developed the design of the study, helped to coordinate data collection, performed statistical analysis, and drafted the manuscript.  BN provided statistical consultation for the analysis and assistance in design of the study.  SCB coordinated all aspects of data collection and maintenance.  SIB provided statistical consultation for the analysis and assistance in interpreting results.  ASA provided consultation for study design and procedures, as well as manuscript preparation.   DSW provided oversight and guidance for the entire research, including design and implementation of the study and evaluation of results. 

## Supplementary Material

Additional file 1 Nutrition and Physical Activity Self-Assessment for Child Care. This self-assessment instrument is used by child care facilities to assess their nutrition and physical activity environments. Click here for file
