# Supplementary material for: Reliability and validity of a nutrition and physical activity environmental self-assessment for child care
Source: Int J Behav Nutr Phys Act. 2007 Jul 5;4:29. doi: 10.1186/1479-5868-4-29 (PMC1934916; doi:10.1186/1479-5868-4-29)
Supplement: Additional file 1 — Nutrition and Physical Activity Self-Assessment for Child Care. This self-assessment instrument is used by child care facilities to assess their nutrition and physical activity environments. [file 1479-5868-4-29-S1.pdf]

**D.** Staff restrict active play time for children who misbehave:	☐ Often	☐ Sometimes	☐ Never	☐ Never and provide more active play time to reward
**E.** Children are seated (excluding nap time) more than 30 minutes at a time:	☐ 1 or more times each day	☐ 3-4 times per week	☐ 1-2 times per week	☐ Less than one time per week or never

(PA2) TV Use and TV Viewing

**A.** Television use consists of the:	☐ TV turned on most of the day, including meal time, everyday	☐ TV turned on for part of the time, most days	☐ TV turned on everyday for part of the time, some days	☐ TV used rarely and only for viewing educational programs
**B.** Children are allowed to watch TV, videos or play video games:	☐ Throughout the day	☐ Once a day	☐ 2-4 times per week	☐ 1 time per week or less, usually for educational use only

(PA3) Play Environment

**A.** Fixed play equipment (swings, slides, climbing equipment, overhead ladders) is:	☐ Unavailable at our site	☐ Swing sets (or one type of equipment) only available	☐ Different equipment available that suits most children	☐ Wide variety of equipment available and accommodates needs of all children
**B.** Safety checks on equipment occur:	☐ Only when equipment is installed	☐ 1 time per year	☐ 1 time per month	☐ 1 time per week
**C.** Portable play equipment that stimulates a variety of gross motor skills (wheel toys, balls, tumbling mats) consists of:	☐ Little variety and children must take turns	☐ Some variety but children must take turns	☐ Good variety but children must take turns	☐ Lots of variety for all children to use at the same time
**D.** When weather is not suitable to go outdoors, indoor play space is available:	☐ For quiet play	☐ For very limited movement (jumping and rolling)	☐ For some active play (jumping, rolling and skipping)	☐ For all activities, including running

(PA4) Supporting Physical Activity

**A.** During active (free) play time staff:	☐ Rarely or never join children in active play (mostly sit or stand)	☐ Sometimes join children in active play	☐ Often or always join children in active play	☐ Often or always join children in active play and make positive statements about the activity

**B.** Staff show visible support for physical activity by:	☐ No posters, pictures, or books about physical activity displayed	☐ A few posters, pictures, or or books about physical activity displayed in a few rooms	☐ Posters, pictures, or books about physical activity are displayed in most rooms	☐ Posters, pictures, or books about physical activity are displayed in every room

(PA5) Physical Activity Education for Children, Parents, and Staff

**A.** Training opportunities are provided for staff in physical activity:	☐ Rarely or never	☐ Less than 1 time per year	☐ 1 time per year	☐ 2 times per year
**B.** Physical activity training is provided by qualified professional (nurse, athletic trainer, doctor):	☐ Rarely or never	☐ Less than 1 time per year	☐ 1 time per year	☐ 2 times per year
**C.** Staff provide physical activity education for children:	☐ Rarely or never	☐ 1 time per month	☐ 2-3 times per month	☐ 1 time per week
**D.** Physical activity education is offered to parents (workshops and activities):	☐ Rarely or never	☐ Less than 1 time per year	☐ 1 time per year	☐ 2 times per year

(PA6) Center Physical Activity Policy

**A.** A written policy on physical activity that covers most of the NAP SACC areas is:	☐ Not available	☐ Available but not followed by most staff	☐ Available but followed only by some staff	☐ Available and routinely followed by all staff

For more information about this self-assessment instrument and the NAP SACC project, please visit http://www.napsacc.org

Please use the following citation when referencing this instrument: Ammerman, AS, Benjamin, SE, Sommers, JS, Ward, DS. 2004. The Nutrition and Physical Activity Self-Assessment for Child Care (NAP SACC) environmental self-assessment instrument. Division of Public Health, NC DHHS, Raleigh, NC, and the Center for Health Promotion and Disease Prevention, UNC-Chapel Hill, Chapel Hill, NC.

State of North Carolina • Michael F. Easley, Governor | Department of Health and Human Services • Carmen Hooker Odom, Secretary
Division of Public Health • NC Healthy Weight Initiative
Department of Nutrition • UNC Schools of Public Health and Medicine | UNC Center for Health Promotion and Disease Prevention

Nutrition And Physical Activity Self-Assessment for Child Care

Your Name: ______________________ Date: __________

Child Care Center/Home Name: ______________________

____

Please read each statement or question carefully and check the response that best fits your child care center or home. Your honest responses will help us work with you to build a healthy nutrition and physical activity environment at your center or home.

SECTION I: NUTRITION

(N1) Fruits and Vegetables

**A.** Fruit (not juice) is served:	☐ 2 times per week or less	☐ 3-4 times per week	☐ 1 time per day	☐ 2 or more times per day
**B.** Fruit is served fresh, frozen, or canned in own juice:	☐ Rarely or never	☐ Some of the time	☐ Most of the time	☐ All of the time
**C.** 100% fruit juice is served:	☐ 2 or more times per day	☐ 1 time per day	☐ 2-4 times per week	☐ 1 time per week or less
**D.** Vegetables (not including french fries or fried potatoes) are served:	☐ 2 times per week or less	☐ 3-4 times per week	☐ 1 time per day	☐ 2 or more times per day
**E.** Vegetables served are dark green, red, orange, or yellow in color:	☐ Less than 1 time per week	☐ 1-2 times per week	☐ 3-4 times per week	☐ 1 or more times per day
**F.** Cooked vegetables are prepared with added meat fat, margarine or butter:	☐ 1 or more times per day	☐ 3-4 times per week	☐ 1-2 times per week	☐ Less than 1 time per week

(N2) Fried Foods and High Fat Meats

**A.** Fried or pre-fried meats (chicken nuggets) or fish (fish sticks) are served:	☐ 1 or more times each day	☐ 3-4 times per week	☐ 1-2 times per week	☐ Less than once a week or never
**B.** Fried or pre-fried potatoes (french fries, tater tots, hash browns) are served:	☐ 1 or more times each day	☐ 3-4 times per week	☐ 1-2 times per week	☐ Less than once a week or never
**C.** High fat meats (sausage, bacon, hot dogs, bologna, ground beef) are served:	☐ 1 or more times each day	☐ 3-4 times per week	☐ 1-2 times per week	☐ Less than once a week or never

**D.** Lean meats, (baked or broiled chicken, turkey, or fish) are served:	☐ Less than once a week	☐ 1-2 times per week	☐ 3-4 times per week	☐ 1 or more times per day
**(N3) Beverages**				
**A.** Drinking water is available outside:	☐ Not freely available	☐ Available during designated water breaks	☐ Easily visible and available on request	☐ Easily visible and available for self-serve
**B.** Drinking water is available inside:	☐ Not freely available	☐ Available during designated water breaks	☐ Easily visible and available on request	☐ Easily visible and available for self-serve
**C.** Sugary drinks (Kool-aid™, sports drinks, sweet tea, punches, soda) other than 100% juice are served:	☐ 1 or more times each day	☐ 3-4 times per week	☐ 1-2 times per week	☐ Less than one time per week or never
**D.** Milk served to children ages 2 and older is usually:	☐ Whole or regular	☐ 2% reduced fat	☐ 1% low-fat	☐ Skim or non-fat
**E.** Soda and other soft-drink vending machines are located:	☐ In entrance or front of building	☐ In public areas but not entrance	☐ Out of sight of parents and children	☐ No vending machines on site
**(N4) Menus and Variety**				
**A.** Menus used are:	☐ 1-week cycle	☐ 2-week cycle	☐ 3-week or more without seasonal change	☐ 3-week or more with seasonal change
**B.** Menus include whole grain foods that are high in fiber (whole wheat bread, oatmeal, brown rice, Cheerios™):	☐ 1 time per week or less	☐ 2-4 times per week	☐ 1 time per day	☐ 2 or more times per day
**C.** Weekly menus include a combination of both new and familiar foods:	☐ Rarely or never	☐ Some of the time	☐ Most of the time	☐ All of the time
**D.** Weekly menus include foods from a variety of cultures:	☐ Rarely or never	☐ Some of the time	☐ Most of the time	☐ All of the time
**(N5) Meals and Snacks**				
**A.** When children eat less than half of a meal or snack, the staff help determine if they are full before removing plate:	☐ Rarely or never	☐ Some of the time	☐ Most of the time	☐ All of the time

Ammerman, AS, Benjamin, SE, Sommers, JS, Ward, DS. 2004. The Nutrition and Physical Activity Self-Assessment for Child Care (NAP SACC) environmental self-assessment instrument. Division of Public Health, NC DHHS, Raleigh, NC, and the Center for Health Promotion and Disease Prevention, UNC-Chapel Hill, Chapel Hill, NC.

**B.** When children request seconds, staff help children determine if they are still hungry before serving the requested food:	☐ Rarely or never	☐ Some of the time	☐ Most of the time	☐ All of the time
**C.** Children who are picky eaters (able to eat a food but resisting) are encouraged to try new or less favorite food:	☐ Rarely or never	☐ Some of the time	☐ Most of the time	☐ All of the time
**D.** Sweets or high fat, high salt foods (cookies, cakes, candy, chips, cheese doodles) are served for snacks:	☐ 1 or more times each day	☐ 3-4 times per week	☐ 1-2 times per week	☐ Less than one time per week
**E.** Food is used to reward desired behavior:	☐ All of the time	☐ Most of the time	☐ Some of the time	☐ Rarely or never
**F.** Food is used to control behavior or withheld as punishment:	☐ All of the time	☐ Most of the time	☐ Some of the time	☐ Rarely or never
**(N6) Foods Offered Outside of Regular Meals and Snacks**				
**A.** Guidelines provided to parents for food brought in for holidays or celebrations are:	☐ Not available	☐ Loose guidelines with healthier options encouraged	☐ Written guidelines for healthier options that are not always enforced	☐ Written guidelines for healthier options that are usually enforced
**B.** Holidays are celebrated with mostly healthy foods or with non-food treats like stickers:	☐ Rarely or never	☐ Some of the time	☐ Most of the time	☐ All of the time
**C.** Fundraising consists of selling only non-food items (like wrapping paper, coupon books or magazines):	☐ Rarely or never	☐ Some of the time	☐ Most of the time	☐ All of the time
**(N7) Supporting Healthy Eating**				
**A.** Children and staff sit down together for meals:	☐ Rarely or never	☐ Some of the time	☐ Most of the time	☐ All of the time
**B.** Meals are served family style (children serve themselves with limited help):	☐ Rarely or never	☐ Some of the time	☐ Most of the time	☐ All of the time
**C.** Staff consume the same food and drinks as the children:	☐ Rarely or never	☐ Some of the time	☐ Most of the time	☐ All of the time

Ammerman, AS, Benjamin, SE, Sommers, JS, Ward, DS. 2004. The Nutrition and Physical Activity Self-Assessment for Child Care (NAP SACC) environmental self-assessment instrument. Division of Public Health, NC DHHS, Raleigh, NC, and the Center for Health Promotion and Disease Prevention, UNC-Chapel Hill, Chapel Hill, NC.

**D.** Staff eat or drink less healthy foods (especially sweets, salty snacks, and sugary drinks) in front of the children:	☐ All of the time	☐ Most of the time	☐ Some of the time	☐ Rarely or never
**E.** Staff talk with children about trying and enjoying healthy foods:	☐ Rarely or never	☐ Some of the time	☐ Most of the time	☐ All of the time
**(N8) Nutrition Education for Children, Parents, and Staff**				
**A.** Training opportunities on nutrition are provided for staff:	☐ Rarely or never	☐ Less than 1 time per year	☐ 1 time per year	☐ 2 times per year or more
**B.** Nutrition training is provided by qualified professional (nurse, registered dietitian, doctor):	☐ Rarely or never	☐ Some of the time	☐ Most of the time	☐ All of the time
**C.** Staff provide nutrition education for children:	☐ Rarely or never	☐ 1 time per month	☐ 2-3 times per month	☐ 1 time per week or more
**D.** Nutrition education opportunities are offered to parents (workshops and activities):	☐ Rarely or never	☐ Less than 1 time per year	☐ 1 time per year	☐ 2 times per year or more
**(N9) Nutrition Policy**				
**A.** A written policy on nutrition and food service that covers most of the NAP SACC is:	☐ Not available	☐ Available but not followed by most staff	☐ Available but followed only by some staff	☐ Available and routinely followed by all staff

SECTION II: PHYSICAL ACTIVITY

**(PA1) Active Play and Inactive Time**				
**A.** Active (free) play time is provided to all children:	☐ 15-30 minutes each day	☐ 31-45 minutes each day	☐ 46-60 minutes each day	☐ More than 60 minutes each day
**B.** Structured physical activity (teacher-led) is provided to all children:	☐ Less than 1 time per month	☐ 2-4 times per month	☐ 2-4 times per week	☐ Daily
**C.** Outdoor active play is provided for all children:	☐ 1 time per week or less	☐ 2-4 times per week	☐ 1 time per day	☐ 2 or more times per day

Ammerman, AS, Benjamin, SE, Sommers, JS, Ward, DS. 2004. The Nutrition and Physical Activity Self-Assessment for Child Care (NAP SACC) environmental self-assessment instrument. Division of Public Health, NC DHHS, Raleigh, NC, and the Center for Health Promotion and Disease Prevention, UNC-Chapel Hill, Chapel Hill, NC.
